# Making the most of canopy light: shade avoidance under a fluctuating spectrum and irradiance

**DOI:** 10.1093/jxb/erae334

**Published:** 2024-08-05

**Authors:** Romina Sellaro, Maxime Durand, Pedro J Aphalo, Jorge J Casal

**Affiliations:** Universidad de Buenos Aires, Consejo Nacional de Investigaciones Científicas y Técnicas (CONICET), Instituto de Investigaciones Fisiológicas y Ecológicas Vinculadas a la Agricultura (IFEVA), Facultad de Agronomía, Buenos Aires, Argentina; Organismal and Evolutionary Biology Research Programme, Viikki Plant Science Centre, Faculty of Biological and Environmental Sciences, University of Helsinki, Helsinki, Finland; Organismal and Evolutionary Biology Research Programme, Viikki Plant Science Centre, Faculty of Biological and Environmental Sciences, University of Helsinki, Helsinki, Finland; Universidad de Buenos Aires, Consejo Nacional de Investigaciones Científicas y Técnicas (CONICET), Instituto de Investigaciones Fisiológicas y Ecológicas Vinculadas a la Agricultura (IFEVA), Facultad de Agronomía, Buenos Aires, Argentina; Fundación Instituto Leloir and IIBBA-CONICET. Av. Patricias Argentinas 435, Buenos Aires C1405BWE, Argentina; Siksha O Anusandhan University, India

**Keywords:** Cryptochrome, lightflecks, photosensory receptors, phytochrome, sunflecks, UVR8

## Abstract

In the field, plants face constantly changing light conditions caused by both atmospheric effects and neighbouring vegetation. This interplay creates a complex, fluctuating light environment within plant canopies. Shade-intolerant species rely on light cues from competitors to trigger shade avoidance responses, ensuring access to light for photosynthesis. While research often uses controlled growth chambers with steady light to study shade avoidance responses, the influence of light fluctuations in real-world settings remains unclear. This review examines the dynamic light environments found in woodlands, grasslands, and crops. We explore how plants respond to some fluctuations but not others, analyse the potential reasons for these differences, and discuss the possible molecular mechanisms regulating this sensitivity. We propose that studying shade avoidance responses under fluctuating light conditions offers a valuable tool to explore the intricate regulatory network behind them.

## Teaser

We describe the fluctuations of the light environment to which plants are exposed under natural radiation, how these fluctuations affect growth responses to neighbour cues, and current understanding of the underlying molecular mechanisms.

## Introduction

Fine-grained variation of illumination can be seen as small patches of light or shade on the ground, hence the use of the word ‘fleck’ to describe them. These patches move, so for a given point in space they create variation in time. With time, the use of fleck or ‘lightfleck’ has expanded to include various kinds of temporal variation in the light to which plants and their parts are exposed. This variation in light amount and quality affects the two roles of light for plants: a source of energy and a source of information.

Finding out what are the light cues that plants can perceive, what information they extract from the perceived cues, and how the responses triggered contribute to fitness (or, for crops, to yield) is detective work requiring ingenuity. Clues for this quest are provided by evolutionary theory, detailed knowledge of the environment, and the nature of the responses of the plants, or other organisms (e.g. Dusenbery, 1992; Aphalo and Ballare, 1995). When cues are non-deterministic and time-varying, the task becomes more challenging than when they are assumed constant, as it becomes necessary to decipher how haphazardly perceived cues are integrated and combined in time by plants during the extraction of information. The fast response of growth to artificial lightflecks differing in irradiance and spectrum was first investigated nearly 50 years ago (Morgan and Smith, 1978). Responses to shade have evolved through natural and artificial selection, and have consequences for individual plants, populations, and communities (Ballaré and Pierik, 2017; Huber *et al.*, 2020). As shade is dynamic, and different canopies differ in their dynamic regime (Durand and Robson, 2023), it can be expected that the cues it creates are perceived by plants and contribute to acclimation and adaptation.

We think that this search for understanding mechanism and function (e.g. Casal, 2013) is most likely to succeed by studying in parallel environmental cues, plant responses to the cues, and the mechanisms of perception, transduction, and extraction of information linking cues to responses. Temporal autocorrelations in individual environmental variables as well as cross-correlations, especially those with lag, among different variables can function as information-carrying cues (Aphalo and Sadras, 2022), while temporal integration and ‘memory’ in plants contribute to fitness (Novoplansky, 2016). Thus, when considering time and whole plants, there are some key questions. (i) Does the response to a short duration cue depend on the time within the photoperiod when it is perceived? (ii) Is the response to repeated exposure to a cue linearly, decreasingly, or increasingly accumulative? (iii) Which response predominates when plants perceive a sequence of contradictory cues? We can, and should, ask what the mechanism behind these responses is, and finally find a logical explanation for the involvement of photoreceptors based on the properties of the light environment.

Acknowledging that adaptation and acclimation are the end-result of processes taking place at multiple time scales and contributing to fitness through multiple mechanisms is a first step (Aphalo and Sadras, 2022). Controlled-environment research on plant responses to shade has mostly relied on daylong and end-of-day treatments, implicitly assuming that average light conditions during the photoperiod or at its end are the main drivers of the studied responses. Field research on photomorphogenesis has usually lacked parallel characterization of the fast temporal dynamics of spectral irradiance, in both cases mainly due to technical difficulties.

Lightflecks can be crucial for plants growing in the shade of a canopy, both as the main source of energy for photosynthesis and as a challenge capable of causing damage (Pearcy, 1990). Evolutionary theory thus suggests that plants must have evolved mechanisms to adjust their physiology and morphology in response to the perceived likelihood of future exposure to large and fast changes in photon irradiance, possibly over-riding responses to cues perceived at times of the day when irradiance varies less or more slowly.

Here we review recent advances in the characterization of the temporal dynamics of the light environment of plants and in our understanding about plant responses to flecks.

## Changes in incoming sunlight caused by atmospheric factors

### Solar elevation

Both the spectrum and irradiance above vegetation vary with the position of the sun in the sky.

**Fig. 1.**
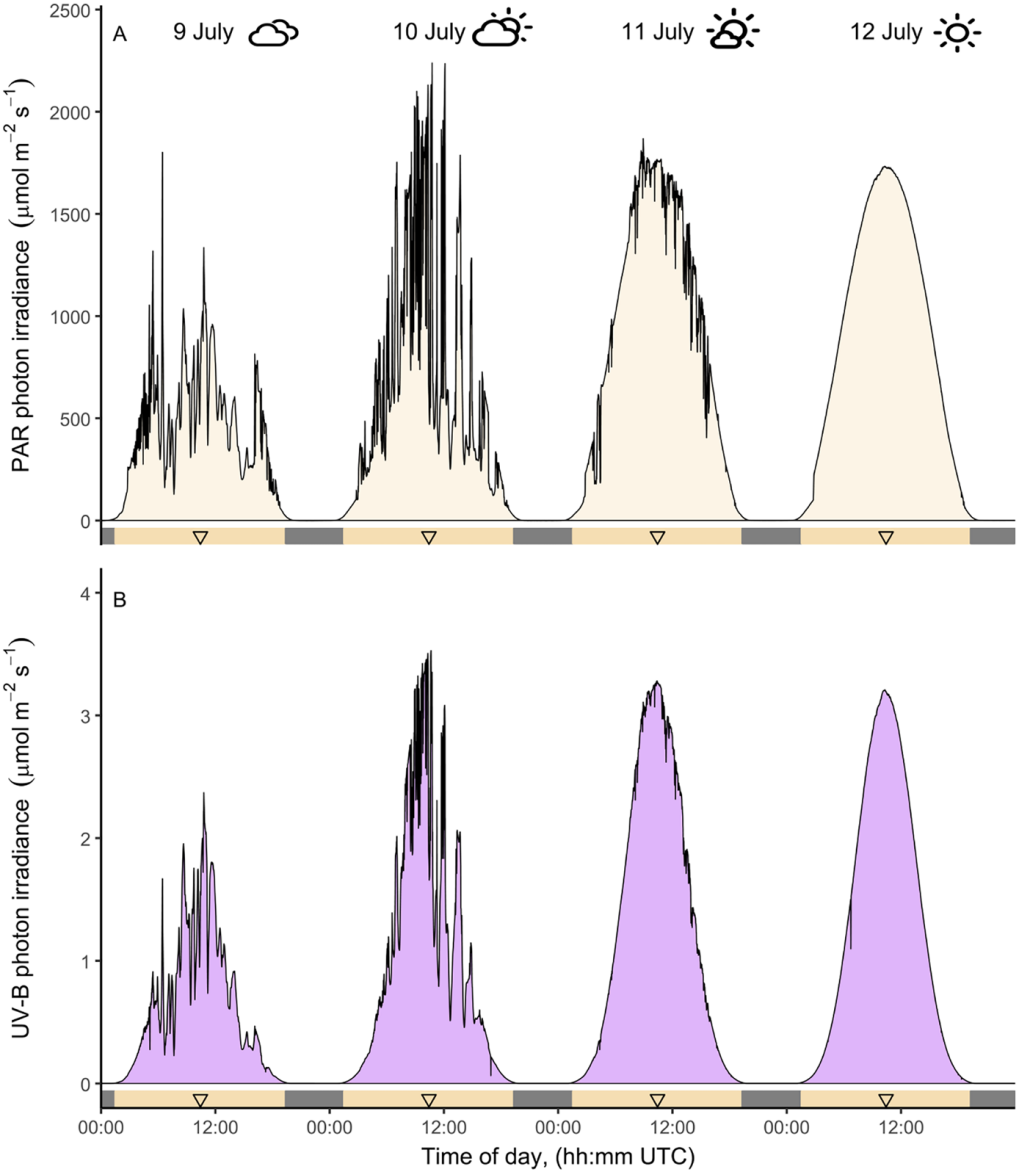
The daily course of irradiance reaching vegetation is affected by cloudiness. Time course of irradiance at Helsinki, Finland, during four consecutive summer days (2023). The lengths of the photoperiods from sunrise to sunset are defined by clear segments in abscissas (18.56–18.40 h), where the triangles indicate local solar noon. Measurements were obtained every 5 s and averaged for each minute. The weather symbols describe sky conditions. The fraction of the time when the sun was 19° above the horizon, and occluded by clouds was: 92% and almost never fully visible (9 July), 55% and at times fully visible (10 July), 1% and occasionally minor effect of clouds (11 July), and 0%, not even partly occluded (12 July). (A) Photosynthetically active radiation (PAR, 400–700 nm). (B) Ultraviolet-B radiation (UV-B, 280–315 nm). Note that the base of the curves is narrower in (B) than in (A). Drawn after Aphalo (2024).

The daily course of irradiance above vegetation is thus variable, and different even between successive days (Fig. 1). It is also different for ultraviolet-B (UV-B) radiation and photosynthetically active radiation (PAR) (Fig. 1) because a lengthening of the path through the atmosphere attenuates UV-B more than PAR. Scattering of sunlight in the atmosphere varies inversely with wavelength (Monteith and Unsworth, 2008). Thus, UV radiation is more diffuse than PAR or far-red light. Visually, daylight scattering appears as a distant haze, especially in the UV range (Supplementary Fig. S1) (Lindfors and Ylianttila, 2016). A similar difference exists between blue and red light, hence the blue colour of the sky.

**Fig. 2.**
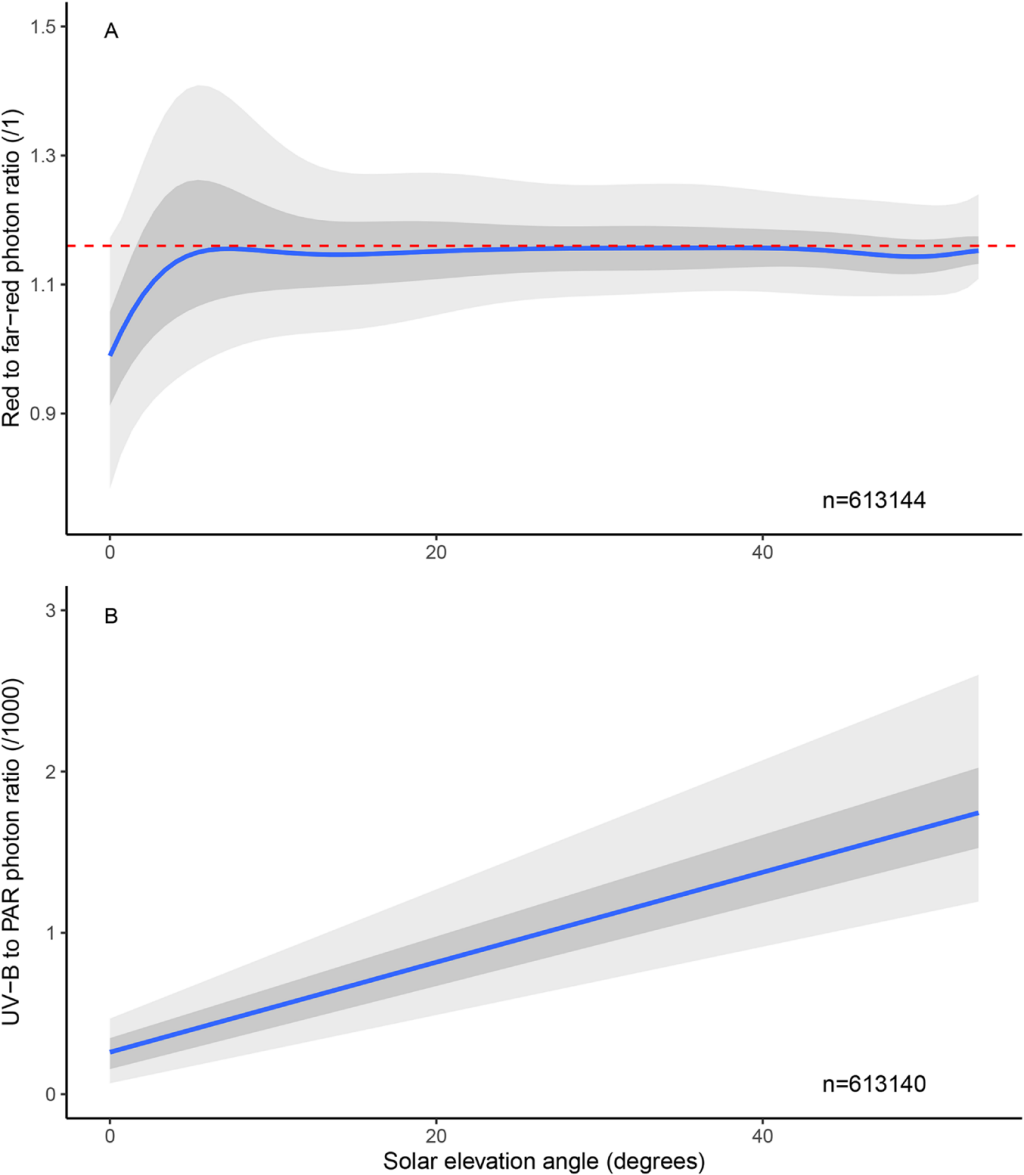
Solar elevation affects the spectral distribution of the incident radiation. Photon ratios as a function of sun elevation during the spring and summer at Helsinki, Finland. (A) Red (655–665 nm)/far-red (725–735 nm) ratio. (B) Ultraviolet-B (280–315 nm)/photosynthetically active radiation (PAR, 400–700 nm) photo ratio. The lines are median regressions, the inner band is limited by the quartiles, and the outer, paler, band encloses 90% of all observations. Data for April to October, 2020–2023, 1 min means from data measured every 5 s. *n*>600 000. Drawn after Aphalo (2024).

The usually assumed red/far-red photon ratio of 1.16 is an approximation, not a constant. In Fig. 2, we see that both the red/far-red and UV-B/PAR photon ratios vary over a range of values depicted as bands. This variation is in part due to seasonal and in part due to geographic changes in solar angle (Kotilainen *et al.*, 2020). This difference in the day course of irradiance means that the UV-B/PAR photon ratio changes with the sun elevation angle (i.e. during each day), with seasons, and with latitude (Fig. 2B). In contrast, the red/far-red photon ratio varies only moderately with sun elevation and mainly at low sun elevations (Fig. 2A) (Kotilainen *et al.*, 2020).

### Variation in the atmosphere

Clouds and aerosols, and gases in the atmosphere modify the daylight spectrum and irradiance through selective absorption and scattering (Supplementary Fig. S2) (Gates, 2003; Monteith and Unsworth, 2008). In addition, spectral radiance, and thus the spectrum of light arriving from different regions of the sky, is also affected by clouds and aerosols (Cordero *et al.*, 2023). Clouds can occlude the solar disk, while much of the sky remains visible. UV-B radiation in daylight is more scattered than PAR; that is, more of it arrives from the sky rather than directly from the sun (Supplementary Fig. S3). Thus, when the sun is occluded, radiation of shorter wavelengths, including UV-B radiation, is attenuated proportionally less than longer wavelengths and the UV-B/PAR and blue/red photon ratios increase (Supplementary Fig. S2). Since UV radiation is more diffuse than PAR or far-red light, the boundaries between sunlight and the shade projected by clouds is more gradual, and shading by clouds is weaker in the UV range than at longer wavelengths (Lindfors and Ylianttila, 2016). Because white clouds strongly reflect sunlight, when the sun is not occluded, they can locally increase irradiance. Thus, the highest instantaneous PAR and UV-B irradiances are not experienced under clear sky conditions but instead under broken clouds (e.g. 12 July compared with 10 July in Fig. 1).

**Fig. 3.**
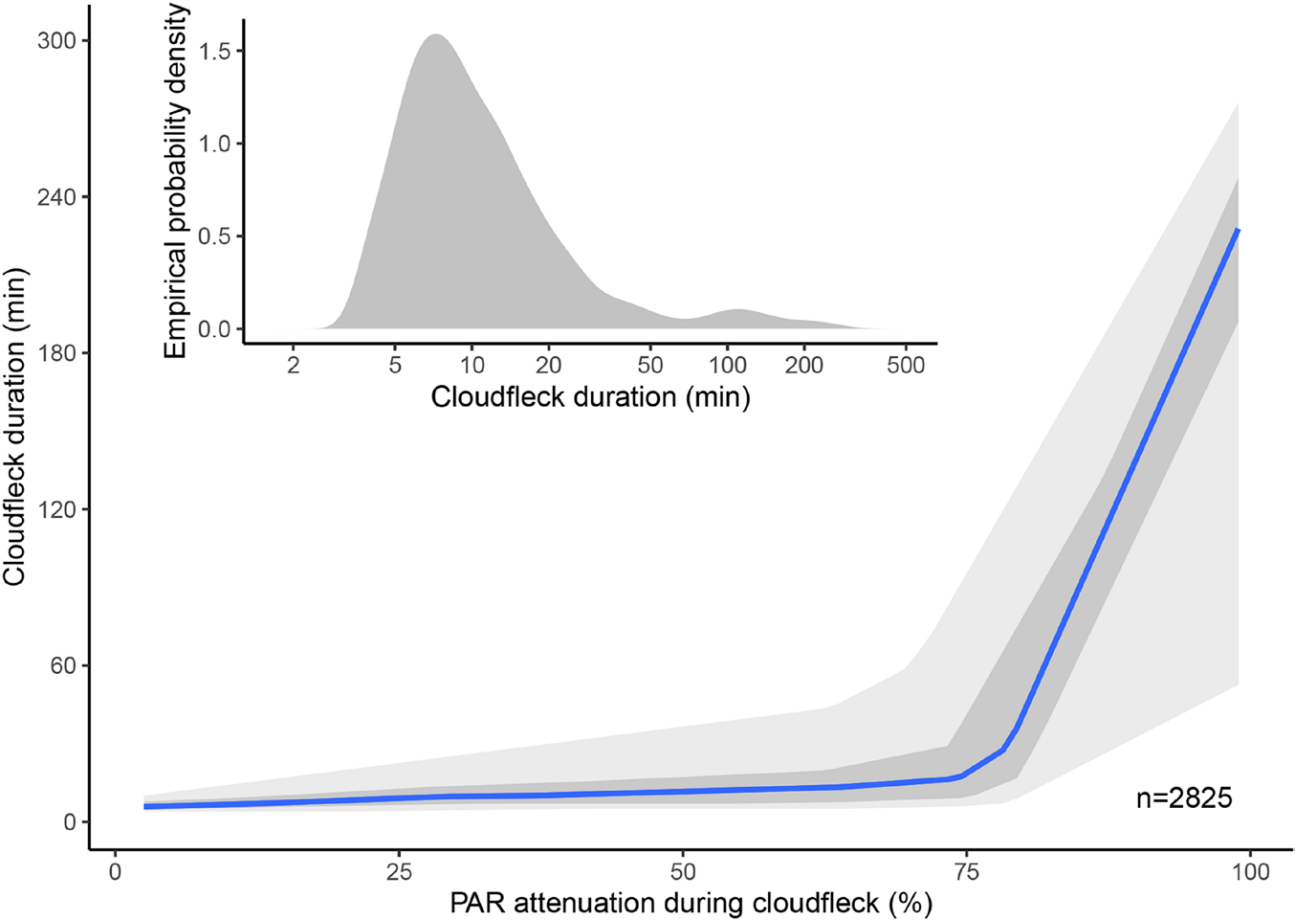
Cloudflecks of longer duration tend to cause stronger light attenuation. Dynamics of light attenuation by clouds during the spring and summer at Helsinki, Finland. Each data point represents a cloudfleck—a transient decrease in irradiance caused by the passing of a cloud. The most common type of cloud cover over Helsinki in the summer is a partly cloudy sky with many white cumulus clouds. Inset, the empirical density distribution function for cloudfleck duration is shown. The lines are median regressions, the inner band is limited by the quartiles, and the outer, paler, band encloses 90% of all observations. Data for 1 May to 30 September 2022 between 10.00 h and 16.00 h local time. *n*>2825. Drawn after Aphalo (2024), using an algorithm previously described (Durand *et al.*, 2021).

Clouds move in the wind, and their moving shade generates cloud flecks on the ground, as can be seen on the mountain at the centre of Supplementary Fig. S1. Depending on the cloud cover and type of clouds, their light attenuation and duration vary (Fig. 3). The darker the shade during a cloud fleck, the more likely it is to last for a longer time. In the case of this example, the most frequent cloud fleck duration was ~7 min (Fig. 3 inset), while occasionally a cloud fleck lasted for several hours. Most cloud flecks lasting for <10 min attenuated PAR by <75% (Fig. 3).

In addition to the well-known effect of ozone in the atmosphere on UV-B irradiance (Graedel and Crutzen, 1993), both ozone and water vapour can slightly modify the red/far-red ratio, especially at low solar elevations (Kotilainen *et al.*, 2020). Some occasional weather events, such as the Saharan dust blowing in the high atmosphere from Northern Africa, can affect the irradiance (up to a 12% decrease in PAR) and the blue/red photon ratio, due to the selective attenuation of short wavelengths by the particles, whilst the effects on the red/far-red ratio are small (Ohde and Siegel, 2013).

### Photoperiod

The length of the photoperiod is deterministic, given by the movements of the Sun and Earth. However, a given daylength occurs twice per year during opposite seasons. Plants and other organisms rely on additional cues, such as temperature, to distinguish Spring from Autumn (Hänninen, 2016). The photoperiod as usually defined is based on visible light. Even if rarely considered as a timing or seasonal cue, UV-B irradiance depends on solar elevation more strongly than PAR; that is, the daily strong UV-B period is shorter than the strong PAR period (Fig. 1).

## Light fluctuation within vegetation canopies

The photosynthetic pigments of green leaves attenuate PAR as radiation penetrates through the canopy layers, causing drops in the red/far-red ratio (Holmes and Smith, 1977) and blue/green ratio (Sellaro *et al.*, 2010). Relatively slight drops in the red/far-red ratio below the values observed for unfiltered sunlight at midday (e.g. 1.1–1.2) can be biologically significant as plants can respond even to far-red light reflected on neighbouring vegetation, which is not projecting shade onto them (Ballaré *et al.*, 1987).

The light environment within canopies varies more than that above them, as additional factors come into play, adding to the variation already present in daylight above the vegetation. At the same time, these new sources of variation can create information-carrying cues distinct from those present in daylight above vegetation.

### Historical perspective

From the shaded plant tissues deep in the understorey to the leaves at the very top of the canopy, all will experience continual fluctuations throughout the day in the amount of radiation incident on the leaf surface. Even minor wind gusts can alter a leaf’s sun-exposed surface area, dynamically influencing both reflected and absorbed radiation.

With the seminal works from Robert W. Pearcy (Pearcy, 1990) and Robin Chazdon (Chazdon, 1988), research on lightflecks surged in popularity in the late 1980s to early 2000s. Lightflecks were initially recognized as crucial for understorey vegetation (herbs and shrubs; Blackman and Rutter, 1946; Chazdon and Field, 1987; Pfitsch and Pearcy, 1989) and tree regeneration (Pearcy, 1983; Leakey *et al.*, 2005), receiving less focus in studies of crops (Norman *et al.*, 1971; Pearcy *et al.*, 1990) or orchards (Lakso and Barnes, 1978). Then, echoing lightflecks, research on light fluctuations quietened down before showing a renewed and rapidly growing interest in recent years (Slattery *et al.*, 2018; Tanaka *et al.*, 2019; Long *et al.*, 2022). This was invigorated by the recognition that light fluctuations were ubiquitous in plant canopies (Way and Pearcy, 2012; Kaiser *et al.*, 2015), and a desire to engineer photosynthesis for improved yields (Long *et al.*, 2015; López-Calcagno *et al.*, 2020; Yoon *et al.*, 2020). This renewed interest highlights the growing recognition of light fluctuations as a critical factor in plant growth, with potential applications for improving crop yields through future engineering efforts.

### A glossary of lightflecks

**Fig. 4.**
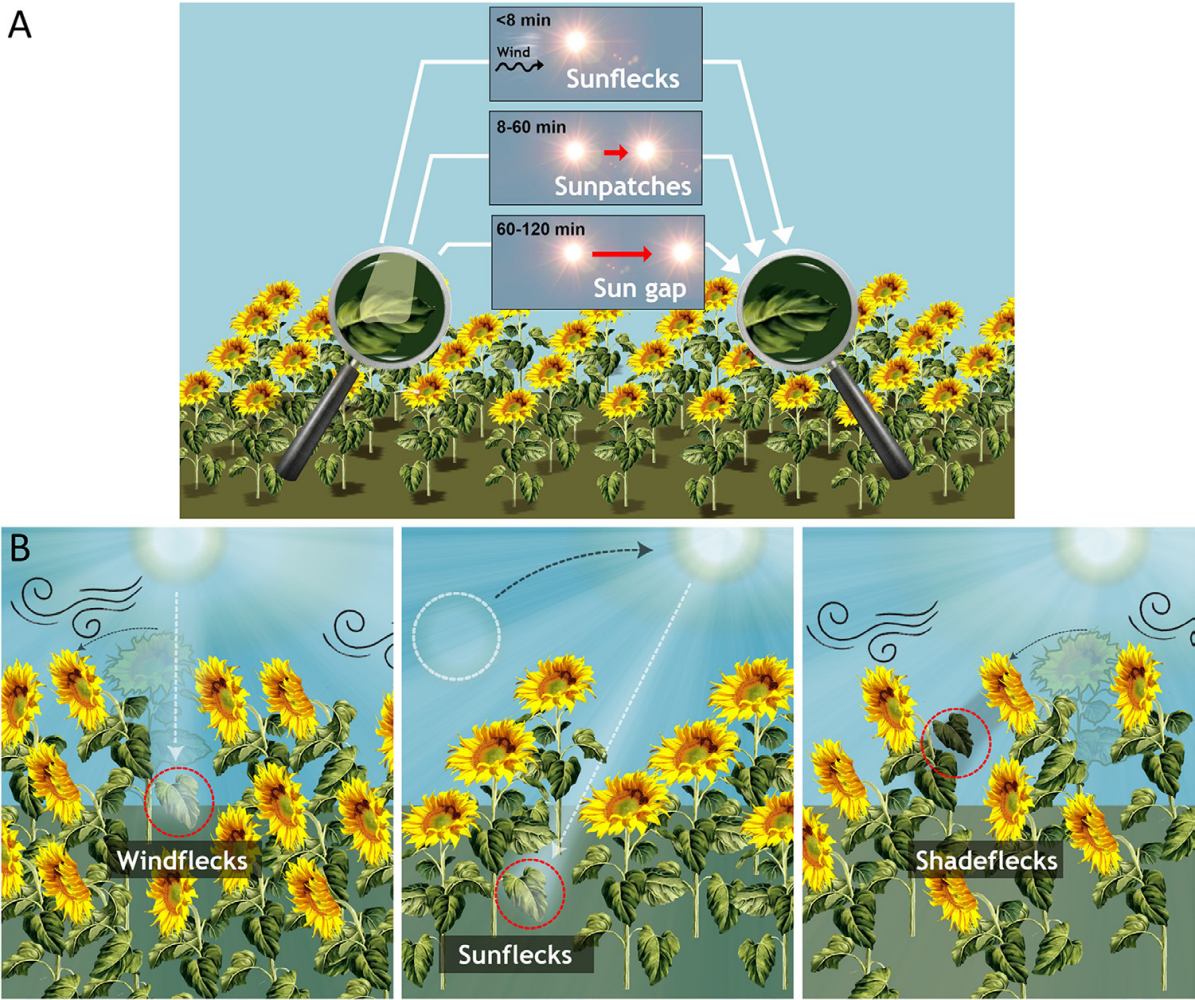
Nomenclature of light fluctuations within plant canopies. (A) Definitions based on the duration of the interruption of shade. (B) Definitions based on the origin of the interruption of shade.

The term ‘sunfleck’, often used to described lightflecks that interrupt shade, has been around for >100 years (McLean, 1919). Since then, many definitions of sunfleck and lightfleck co-exist. Some of these definitions are based on the duration and/or irradiance levels reached during these light fluctuations. For instance, based on duration, a sunfleck lasts <8 min, a ‘sunpatch’ between 8 min and 60 min, and a ‘sun gap’ >2 h (Smith and Berry, 2013) (Fig. 4A). We adhere to this terminology in the sections dealing with plant responses. Other definitions of sunfleck are based on irradiance over a threshold. This can be an absolute threshold, which in different studies has been set at 300 µmol m^–2^ s^–1^ (Roden and Pearcy, 1993), 200 µmol m^–2^ s^–1^ or 100 µmol m^–2^ s^–1^ depending on canopy height (Vierling and Wessman, 2000), or 50 µmol µmol m^–2^ s^–1^ (Miyashita *et al.*, 2012). The threshold has also been defined relative to the irradiance above the canopy (e.g. >70% in Barradas *et al.*, 1998). Finally, thresholds can be defined based on their bell-shaped pattern in time-series of irradiance (Durand *et al.*, 2021), and their duration is best described as an empirical probability distribution.

More recently, lightfleck has been used as a broad term encompassing all fluctuations in light within a canopy (to which we adhere in this review). Lightflecks receive specific names depending on their origin (Fig. 4B). Sunfleck has been redefined as a specific type of lightfleck caused by the sun’s position changing due to Earth’s rotation. These sunflecks can last from minutes to an hour depending on the canopy gap size (Smith and Berry, 2013). ‘Windflecks’ are shorter fluctuations due to plant movements in the wind (typically <1 s; Burgess *et al.*, 2021). Shadeflecks (sometimes considered to fall within or on the opposite end of the definition of lightflecks) are intermittent periods of shade over a background of high irradiance (Pearcy, 1990) and ‘cloudflecks’ are fluctuations due to cloudiness (Fig. 3, typically 15 min, but individually lasting from fractions of minutes to hours; Knapp and Smith, 1988; Kaiser *et al.*, 2018).

### How canopy structure and atmosphere shape light dynamics

The above nomenclature highlights the complexity of light dynamics in nature, which is the product of the Sun’s angle (including both daily and seasonal changes in the Sun’s angle), atmospheric processes (cloudiness, aerosols), and the wind (direction, speed) interacting with the canopy architectural and biomechanical arrangement. As such, both high-frequency fluctuations due to movement of the leaves around the petiole (Roden and Pearcy, 1993), and lower frequency fluctuation due to sun rays passing through gaps in the canopy (Smith and Berry, 2013), happen at the same time. This creates highly convoluted fractal-like temporal dynamics of light. Understanding these light dynamics is crucial for accurately modelling plant growth and photosynthesis in natural environments.

The duration and intensity of lightflecks interrupting shade are intricately linked to canopy structure, influencing the overall light environment experienced by plants. Studies consistently show that longer lightflecks are exponentially less common than shorter ones (Barradas *et al.*, 1998; Vierling and Wessman, 2000; Durand *et al.*, 2021), but longer lightflecks contribute more to total irradiance if they are also more intense, which is itself affected by canopy properties. Broadly, longer lightflecks tend to contribute more to overall irradiance in tall, forested canopies (Pearcy, 1994), because the larger distances between a canopy gap and the location of the lightfleck on a surface produces a large penumbra (area of partial shade) around the lightfleck, with intermediate irradiance (Smith *et al.*, 1989). Thus, delivering high irradiances such as the open sky require longer (and by corollary larger) lightflecks. In contrast, the short canopies of crops species tend to produce a highly contrasted light environment with more intense lightflecks (Durand and Robson, 2023). On this note, previous studies defining lightflecks within the canopy as any irradiance above a subjective threshold would exclude the faster and less intense lightflecks of forest canopies, leading to the conclusion that lightflecks in crops were thought to be shorter than in forests (Pearcy *et al.*, 1990, 1996).

### Challenges in comparing lightfleck data

Canopy architecture dictates light distribution (Falster and Westoby, 2003; Hirose, 2005; Burgess *et al.*, 2017), but few studies have delved into the specific factors determining light fluctuations. Early research comparing lightfleck distribution in plant canopies found that more open canopies produce light fluctuations that are more intense, of longer duration (Chazdon, 1988; Pearcy *et al.*, 1990), and more frequent (Vierling and Wessman, 2000; Miyashita *et al.*, 2012). This resulted from either shorter tree height (Barradas *et al.*, 1998), smaller leaf area (Chazdon and Pearcy, 1991), or measurements at reduced depth within a canopy (Pearcy *et al.*, 1990). However, these comparisons are hampered by methodological inconsistencies. For instance, measurements were made at various frequencies across studies, which can mask smaller lightflecks (see Chazdon, 1988) and affect the overall properties assigned to the lightflecks (Durand *et al.*, 2021). Moreover, measurements in forests are often performed on the forest floor, whereas in crops they are usually done within the canopy (e.g. Durand and Robson, 2023), adding a layer of complexity to comparing studies.

The use of thresholds of irradiance to define a lightfleck arbitrarily excludes smaller fluctuations. Even though it was previously thought as the best way to accurately define lightflecks (Chazdon, 1988), Smith *et al.* (1989) described how the size of a gap in the canopy and its distance to the surface on which the lightfleck is produced are related to the area of the penumbra. A corollary to this is that taller and denser canopies will produce lightflecks with more penumbra, eventually not passing the pre-defined threshold. These smaller lightflecks, often excluded by arbitrary thresholds, may significantly affect understorey leaves due to their rapid light saturation and limited ability to utilize full sun irradiance (Earles *et al.*, 2017; Durand *et al.*, 2022). Future studies employing standardized methodologies across different canopy structures are crucial for a comprehensive understanding of lightfleck dynamics. Sharing of raw irradiance time-series data would facilitate reanalysis and comparisons among studies.

### Spectral changes during lightflecks

**Fig. 5.**
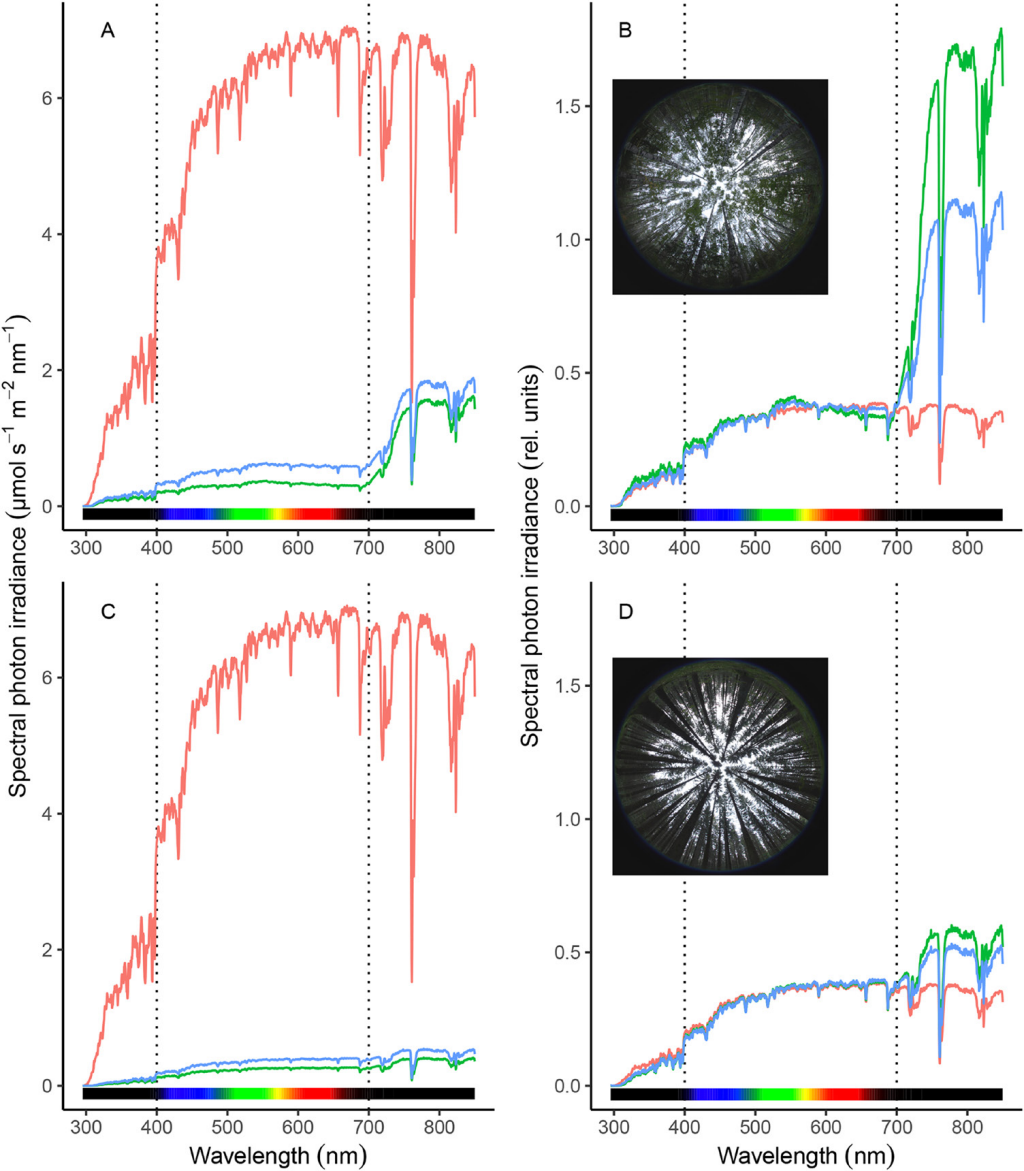
Spectral photon distribution of the radiation within plant canopies. Spectral irradiance in the understorey (shade, green line; sunfleck, blue line) of silver birch (A and B, *Betula pendula*) and Norway spruce (C and D, *Picea abies*) forests, and in a large opening nearby (red line). Measured close to solar noon. Redrawn after Durand and Robson (2023). Spectral photon irradiance is shown in absolute values (A, C) or re-scaled to equal a PAR of 100 µmol m^–2^ s^–1^ (B, D).

Along with changes in irradiance, lightflecks also produce changes in the spectral composition of sunlight within the canopy (Fig. 5). These spectral changes are quite variable (Hartikainen *et al.*, 2018; Hovi and Rautiainen, 2020), and depend on leaf structure, pigment composition (both affecting optical properties; Gates *et al.*, 1965), and the arrangement of canopy elements such as leaf angle and area (Asner, 1998). In general, the spectrum during lightflecks is intermediate between that above the canopy and that in full shade.

Typically, lightflecks are enriched in blue and red light compared with the surrounding shade because leaves preferentially absorb in these wavelengths (Fig. 5; Liu and van Iersel, 2021). They have a lower blue/red ratio and higher red/far-red ratio (Navrátil *et al.*, 2007; Hertel *et al.*, 2011; Durand *et al.*, 2021; Durand and Robson, 2023). Moreover, the UV/PAR ratio often decreases during a transition from shade to lightfleck (Flint and Caldwell, 1998; Hartikainen *et al.*, 2018; Burgess *et al.*, 2021; Durand *et al.*, 2021; Durand and Robson, 2023).

Assessing rapid (<1 s) changes of irradiance in multiple canopies, Durand and Robson (2023) found that each canopy species studied produced a unique change in spectral composition. The UV/PAR ratio also generally declines with depth in the canopy (Yang *et al.*, 1993; Grant, 1997; Deckmyn *et al.*, 2001; Burgess *et al.*, 2021), but this feature is canopy specific (Fig. 5). Still, separate measurements of shade and lightfleck spectral composition along the canopy vertical gradient are extremely rare. Considering that, compared with direct sunlight, diffuse radiation is enriched in short wavelengths (UV, blue) because of their higher scattering probability (Flint and Caldwell, 1998), changes in spectral composition could differ during a lightfleck depending on the depth in the canopy at which it occurs. Similarly, lower solar elevations induce higher proportions of shortwave radiation to be scattered toward space, thus making the radiation above the canopy depleted in UV and blue but enriched in red (Endler, 1993). Overall, changes in the red/far-red ratio are the largest shift in spectral composition between lightflecks and shade (Fig. 5B), but the spectral composition of a lightfleck is highly dynamic, influenced by its location within the canopy, time of day, and the specific canopy structure.

## Light cues as a source of information to control morphogenesis

### Shade avoidance

Light is a key regulator of plant morphology, influencing processes such as seed germination, seedling de-etiolation, and photoperiodic flowering. Here, we will focus on its role in shade avoidance. Several shade-intolerant plant species initiate a suite of shade-avoidance responses upon exposure to shade or even the far-red light reflected from neighbouring plants (without experiencing direct shading, Ballaré *et al.*, 1987). These responses aim to minimize both current and future shading. These strategies encompass the following (Casal and Fankhauser, 2023). (i) Outgrowing competitors: this involves vigorous elongation of stems and petioles, along with hyponastic leaf growth (curving leaves upwards) to reach above neighbouring plants. (ii) Horizontal foliage displacement: asymmetric growth or branching allows the plant to strategically position its leaves towards better-lit areas. (iii) Shade-induced resource allocation: growth of leaves and branches in shaded areas is inhibited, often accompanied by accelerated senescence to redirect resources towards more favourable light conditions. (iv) Phenological adjustments: plants may modulate the timing of flowering and seed germination to avoid periods of intense competition with neighbouring vegetation.

### Kinetics of shade-avoidance responses under dynamic light conditions

While some shade-avoidance responses, such as branching or flowering, are evident after days, the stem reacts much faster to shade cues. Studies in mustard (*Sinapis alba*) and Arabidopsis seedlings (Morgan *et al.*, 1980; Child and Smith, 1987; Cole *et al.*, 2011) reveal rapid responses. Following a brief lag (10 min or 45 min), exposure to a low red/far-red ratio (indicating shade) triggers a surge in stem elongation, peaking at 20 min or 150 min. This is followed by a temporary slowdown and a final acceleration leading to sustained growth beyond 100 min or 230 min (where shorter and longer times correspond in each case to mustard and Arabidopsis, respectively). Notably, mustard exhibits a faster initial growth rate compared with Arabidopsis, which shows a higher rate during the second growth phase (Morgan *et al.*, 1980; Child and Smith, 1987; Cole *et al.*, 2011). Under persistent shade, the response magnitude can further increase (Pucciariello *et al.*, 2018).

In mustard, brief exposures to low red/far-red ratios lasting less than the lag phase trigger a temporary surge of internode growth after the shade disappears. However, longer exposures leading to the second phase result in a reversal, with growth returning to pre-stimulation levels within 16 min upon exposure to a high red/far-red ratio (sunlight) (Morgan *et al.*, 1980; Child and Smith, 1987).

In sparse canopies, far-red light reflected on neighbours can propagate horizontally, reaching the stem of neighbours before the leaves are shaded and the photosynthetic capacity becomes compromised (Ballaré *et al.*, 1987; Casal, 2013). In mustard, exposure of the growing internode itself to the low red/far-red ratios is enough to elicit these rapid and reversible responses (Morgan *et al.*, 1980; Child and Smith, 1987). However, if the leaves also receive low red/far-red ratios for at least 3 h, the growth of the mustard stem remains elevated for up to 24 h after the termination of the neighbour cue (Casal and Smith, 1988). Therefore, there is a correlation between the risk of limitation by light available for photosynthesis and the persistence of the shade-avoidance response. Unlike mustard, Arabidopsis seedlings show a faster return to normal growth rate after shade removal (Pucciariello *et al.*, 2018).

As noted in the previous paragraph, the perception of the cues from neighbours is not limited to the organ that ultimately responds to these cues. The cotyledons perceive low red/far-red ratios in young Arabidopsis seedlings (Procko *et al.*, 2014), and the tip of the leaf serves the same function at the rosette stage (Michaud *et al.*, 2017; Pantazopoulou *et al.*, 2017; Küpers *et al.*, 2023). It is tempting to speculate that the foliage could contribute to integrate spatial heterogeneity of the complex canopy environments over a wider area.

Arabidopsis responds to the initial cues from neighbours throughout the day (Cole *et al.*, 2011). However, distant neighbours may cast shade for short periods daily due to specific sun angles. These repeated shade events, even those lasting 2 h, are ineffective if they occur consistently in the morning and are followed by a prolonged sun (sungap) during the rest of the photoperiod (Sellaro *et al.*, 2012). This suggests that plants can become desensitized to low-level, repetitive shade signals in the morning.

As described above, natural canopies experience sunflecks and sunpatches interrupting shade. Since the occurrence of sunpatches depends on the combination of solar angles and the position of gaps within the canopy, they are typically repeated every day. These interruptions of shade can severely reduce the magnitude of shade-avoidance responses (Sellaro *et al.*, 2011; Moriconi *et al.*, 2018). Even low-frequency sunflecks of intermediate duration (2 min sunflecks every 8 min) can lessen shade avoidance (Belmonte *et al.*, 2024, Preprint). Although brief, the light input during sunflecks can contribute significantly to photosynthesis (Pearcy, 1990; Kaiser *et al.*, 2018; Morales and Kaiser, 2020). However, sunflecks also represent a risk because shade-acclimated tissues become suddenly exposed to strong light (Pearcy, 1990). Thus, the ability to convey sunfleck information to the control of shade avoidance could be important to optimize their magnitude.

In summary, the strength of shade-avoidance responses is influenced by the extent of reduction in the red/far-red ratio and irradiance and the temporal features of the neighbour cues. Persistent shade leads to a stronger response, while brief shade events are less effective. Stem growth exhibits a continuous response, reacting rapidly to shade cues. Plants commit to a stronger response under persistent shade with minimal sunlight interruptions. Conversely, brief, suboptimal shade events lose effectiveness when repeated daily. This highlights the crucial role of shade duration in regulating the magnitude of shade-avoidance responses.

### The effects of changes in incoming sunlight

Whilst plants can respond to changes in the light environment caused by neighbouring vegetation, whether they are affected by changes in incoming sunlight above the canopy is poorly understood, except for the well-established responses to photoperiod. Some of the changes in irradiance and spectral composition of the incoming light could be confounded with neighbour cues, such as the drop in irradiance and red/far-red ratio that occurs at the extremes of the natural photoperiod (Figs 1, 2). At least tillering in grasses is unaffected by these drops (Casal *et al.*, 1990).

## Perception of the transitions between sunlight and shade, and vice versa

### Photoreceptor activity under sunlight

**Fig. 6.**
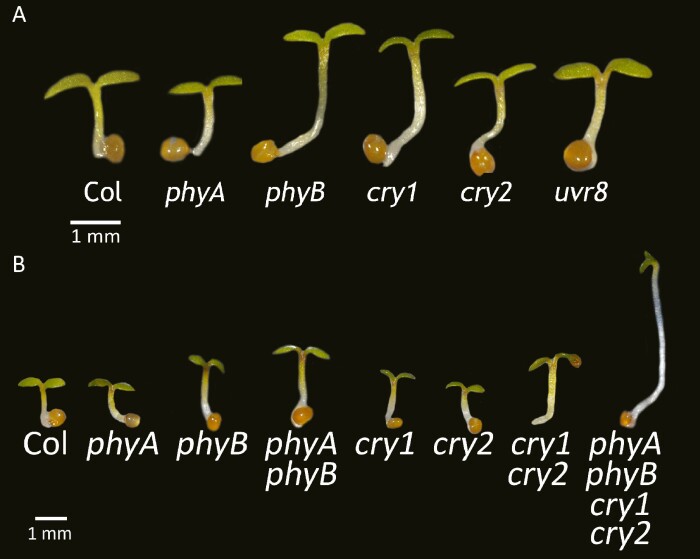
Multiple photosensory receptors are active under sunlight, but the repression of shade avoidance only requires phyB and cry1. (A) Single *phyA*, *phyB*, *cry1*, *cry2*, and *uvr8* mutants compared with the wild type. Only the *phyB* and *cry1* mutations showed a phenotype (Mazzella and Casal, 2001). (B) The comparison of the double *phyA phyB* and *cry1 cry2* mutants and the quadruple *phyA phyB cry1 cry2* mutant with the wild type and their single mutants reveals that phyA and cry2 are active under sunlight (Mazzella and Casal, 2001). Seedlings of *Arabidopsis thaliana* were grown outdoors in plastic boxes as described (Moriconi *et al.*, 2018).

The set of plant photosensory receptors covers a wide range of wavelengths. UV-B is perceived by UV RESISTANCE LOCUS 8 (UVR8) (Podolec *et al.*, 2021). UV-A and blue light are perceived by cryptochromes (crys) (Wang and Lin, 2020), phototropins (Christie, 2007), and the LOV-domain/F-box flavoproteins including ZEITLUPE, the flavin-binding, kelch repeat, F-box 1, and LOV KELCH PROTEIN 2 (Ito *et al.*, 2012). Red and far-red light are perceived by phytochromes (phys) (Burgie and Vierstra, 2014). The spectrum of the polychromatic light and the spectrum of the photoreceptor jointly determine the contribution of the photoreceptors to responses and, for this reason, experiments under natural radiation revealed a role for UVR8 in the perception of UV-A2 (315–340 nm) (Rai *et al.*, 2019, 2020). Plant photosensory receptors activated by high irradiance and sunlight spectrum repress shade-avoidance responses (Casal and Fankhauser, 2023). phyB and cry1 rank as the most crucial photoreceptors repressing shade avoidance under sunlight (Hernando *et al.*, 2021). This concept is illustrated by the phenotype of several photoreceptor mutants under sunlight, where only the *phyB* and *cry1* single mutants exhibit elongated hypocotyls, indicative of a partially released shade-avoidance response (Fig. 6A) (Mazzella and Casal, 2001).

**Fig. 7.**
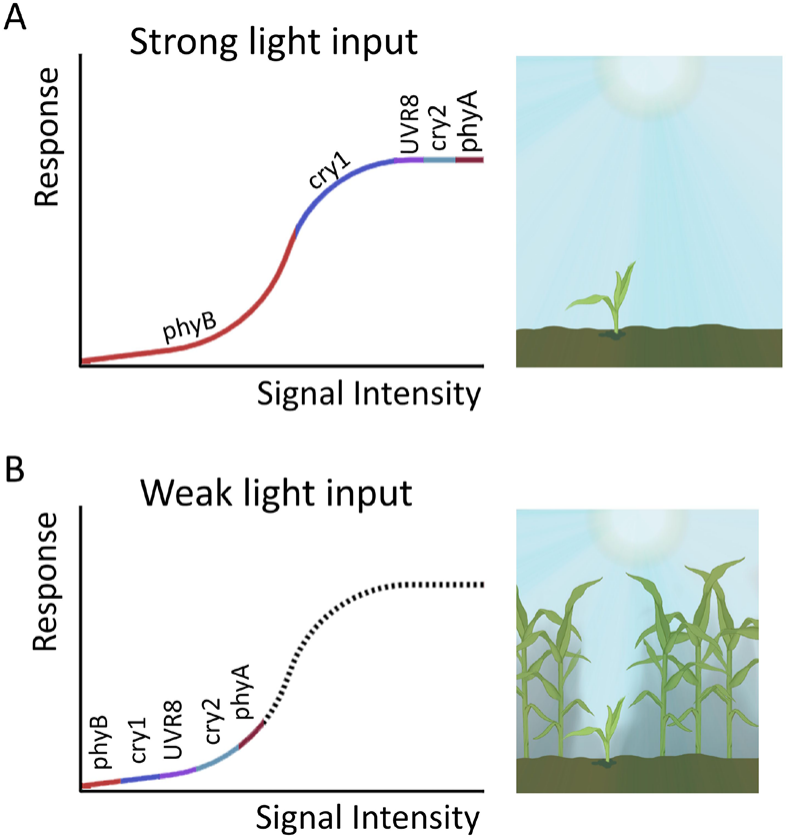
The combined contribution of the activity of multiple photosensory receptors depends on the light input. (A) Under strong light inputs, the contribution of different photoreceptors tends to be redundant. Loss-of-function mutation of some of these photoreceptor genes can reduce the magnitude of their overall signal within a range where this has little impact on the growth response. This feature could generate buffering capacity against non-informative fluctuations of the light environment outside the canopy. (B) Under weak light inputs, the contribution of different photoreceptors tends to be mutually dependent (synergic). Loss-of-function mutation of some of these photoreceptor genes can reduce the magnitude of their overall signal below the minimum required to elicit a response. This feature could enable responses to the brief interruptions of shade caused by sunflecks.

While other single photoreceptor mutants lack a clear phenotype under sunlight, this does not imply inactivity. Glasshouse experiments reveal that the *phyA phyB* double mutant is taller than the *phyB* mutant, and the *cry1 cry2* double mutant is taller than *cry1* (Fig. 6B) (Mazzella and Casal, 2001). This genetic pattern suggests functional redundancy among the photoreceptors. Furthermore, even the *phyA phyB cry1 cry2* quadruple mutant retains some inhibition of hypocotyl growth under sunlight (Mazzella and Casal, 2001; note the partially unfolded cotyledons in the quadruple mutant, Fig. 6B). This indicates that besides phyA, phyB, cry1, and cry2, other photoreceptors might contribute, although their action becomes dispensable in the presence of the main players. Redundancy among photosensory receptors under strong light inputs could help reduce the impact of fluctuations of incoming radiation, while maintaining nearly optimal levels of physiological output (Fig. 7).

### Mechanism for shade perception by phyB

Shade reduces the activity of key shade-avoidance repressors, phyB and cry1. phyB exists in two forms: Pr (absorbing red light) and Pfr (absorbing far-red light) (Burgie and Vierstra, 2014). These forms mutually interconvert upon light absorption. Sunlight’s high red/far-red ratio maintains elevated levels of the Pfr–Pfr dimer (Sellaro *et al.*, 2019), the active conformer of phyB (Klose *et al.*, 2015), which represses shade avoidance. phyB not only detects the shift in red/far-red light ratio caused by shade, but also senses the overall decrease in irradiance (Trupkin *et al.*, 2014; Klose *et al.*, 2015). This dual sensitivity is crucial for shade avoidance. In classical experiments studying the inhibition of hypocotyl growth that occurs when dark-grown seedlings are transferred to continuous red light (a transition called de-etiolation), the response mediated by phyB is clearly fluence rate dependent. This dependency is caused by the spontaneous de-stabilization of Pfr and its reversal to Pr, a reaction called thermal reversion (Klose *et al.*, 2015, 2020). This is also called dark reversion (because, in contrast to the photo-transformations between Pr and Pfr, it does not require light), but the latter terminology generates confusion because it leads to thinking that it only occurs in the dark, which is not true (Klose *et al.*, 2020). The interplay between photoconversion and thermal reversion generates irradiance dependency of phyB activity because part of the Pfr formed by light goes back to Pr, making additional photons necessary to re-establish Pfr (Klose *et al.*, 2015). Since de-etiolation experiments typically involve irradiances that are much weaker than those experienced under sunlight, a role for thermal reversion in shade avoidance normally had not been considered. However, in plants that have completed de-etiolation, the nuclear condensates of phyB, which correlate with phyB activity, change not only when the red/far-red ratio is reduced but also when irradiance is reduced, indicating that phyB is able to perceive the reduction in irradiance (Trupkin *et al.*, 2014). Later, *in vitro* experiments revealed that thermal reversion of phyB is faster than previously thought (Legris *et al.*, 2016) and therefore can compete with the photochemical reactions up to a certain light level, where photochemical reactions are so fast that the impact of thermal reversion becomes fully diluted (Sellaro *et al.*, 2019). While thermal reversion of phyB is less influential under full sunlight, it becomes more relevant in shade or at the fringes of the day/night cycle, allowing plants to perceive reductions in irradiance (Sellaro *et al.*, 2019). Prolonged shade reduces the nuclear levels of phyB, helping to reinforce avoidance responses (Pucciariello *et al.*, 2018).

### Mechanism for shade perception by cry1

cry1 activity is highly dependent on irradiance. In their dark, inactive state, crys are monomers, and the conformational changes induced by light absorption facilitate cry homo-oligomerization, which increases the affinity of the photoreceptor for its interacting partners (Wang and Lin, 2020). The low levels of UV-A and blue light present under shade conditions are predicted to cause monomerization; effectively removing the suppressive effect of cry1 on shade-avoidance responses (Sellaro *et al.*, 2010; Keller *et al.*, 2011; Keuskamp *et al.*, 2011). While green light activates cry1, its presence alongside blue light reduces cry1’s overall activity (Bouly *et al.*, 2007). This interplay between blue and green light contributes to shade avoidance, as the shade environment typically has a lower blue/green ratio compared with sunlight (Sellaro *et al.*, 2010).

### PhyA: opposing phyB and countering excessive shade avoidance

phyB and phyA have opposite responses to shade. Similarly to all other phys, phyA has two forms, Pr and Pfr, but its dependence on irradiance and light quality is more complex (Rausenberger *et al.*, 2011). Pr present in the cytosol must absorb red light to be transformed to Pfr and interact with the proteins able to transport it to the nucleus. Once in the nucleus, Pfr must absorb far-red light to be released from its carrier protein, and the resulting Pr must absorb red light to finally produce Pfr in its subcellular site of action (Rausenberger *et al.*, 2011). Then, under natural radiation, phyA operates as a sensor of red plus far-red radiation and its activity gradually decreases with moderate shade (Sellaro *et al.*, 2010). In addition, phyA activation requires both red and far-red light. Thus, while low red/far-red ratios of very deep shade reduce phyB activity and promote shade avoidance, they actually enhance the activity of phyA, which works against excessive shade avoidance (Yanovsky *et al.*, 1995; Casal *et al.*, 2014; Song *et al.*, 2020). In summary, high irradiances with low red/far-red ratios are optimal for phyA activity, and increasing shade has contrasting effects on these two features of the light environment. In laboratory experiments involving far-red light added to a background of white light to simulate neighbour cues, the effect of phyA in repressing shade avoidance can be exaggerated. In fact, the seedlings receive low red/far-red ratios without the concomitant reduction in irradiance that would occur under actual shade.

### Perception of sunflecks and sunpatches

Modelling suggests that the level of active phyB is highly responsive to short, frequent sunflecks, involving transitions from shade to light and back to shade again within seconds (Sellaro *et al.*, 2019). Further research is needed to investigate whether the effects mediated by phyB under such fluctuating light conditions actually correlate with the integral of active phyB over time.

Under sunlight, phyB and cry1 are the primary photoreceptors that suppress shade-avoidance responses in plants (Fig. 6). While other photoreceptors such as UVR8, phyA, and cry2 are also active, their roles are less crucial in this scenario (Mazzella and Casal, 2001; Moriconi *et al.*, 2018). However, when plants experience prolonged shade with only occasional sunflecks or patches of sunlight, the situation changes. Under these limited light interruptions, photoreceptors such as UVR8, phyA, and cry2 gain significance, working together (synergistically) to counteract shade-avoidance responses (Sellaro *et al.*, 2011; Moriconi *et al.*, 2018; Belmonte *et al.*, 2024, Preprint). In essence, the dominance shifts from phyB and cry1 to a combined effort by multiple photoreceptors when plants encounter brief shade interruptions, where the light input is weak (Fig. 7).

Despite its negligible hypocotyl growth phenotype under sunlight, UV-B perceived by UVR8 effectively reduces the magnitude of shade avoidance when direct light penetrates through gaps in the canopy, interrupting shade (Moriconi *et al.*, 2018; Belmonte *et al.*, 2024, Preprint). When exposed to UV-B radiation, UVR8 rapidly changes from a dimer to a monomer (Rizzini *et al.*, 2011). This change acts as a switch, favouring UVR8 nuclear accumulation and activity (Podolec *et al.*, 2021). In plants exposed to changing light conditions, the proportion of UVR8 dimers and monomers reflects the light environment. Shade environments establish a higher proportion of dimers, while unfiltered sunlight triggers an immediate conversion of dimers to monomers, lowering the ratio (Findlay and Jenkins, 2016; Moriconi *et al.*, 2018). Once sunlight exposure ends, the ratio returns to its shade-induced levels (Moriconi *et al.*, 2018).

The increase in irradiance experienced under lightflecks could be the factor enhancing the activity of phyA towards repressing shade avoidance (Sellaro *et al.*, 2011; Belmonte *et al.*, 2024, Preprint).

### Building sensitivity: the cumulative effect of repeated sunflecks

Sunflecks of relatively long duration often reach a higher peak of irradiance, and therefore they can provide a significant proportion of the radiation for photosynthesis despite their low frequency. Plants can respond to 2 min sunflecks separated by 6 min shade despite the fact that a single 2 min interruption of shade would be insufficient on its own to trigger a measurable repression of shade avoidance (Belmonte *et al.*, 2024, Preprint). However, repeated sunflecks cause changes in the activities of the photoreceptors that increase their sensitivity to subsequent sunflecks, or even to the weak light input experienced under shade between successive sunflecks. A single 2 min sunfleck induces UVR8 monomerization. However, it is not enough for UVR8 to reach the nucleus and become fully functional (Belmonte *et al.*, 2024, Preprint). Even if infrequent, repeated stimulation induces the accumulation of UVR8 in the nucleus, increasing the sensitivity to UV-B (Belmonte *et al.*, 2024, Preprint). Similarly, repeated sunflecks increase the nuclear abundance of cry1, its apparent stage of aggregation, and the sensitivity to blue light (Belmonte *et al.*, 2024, Preprint).

## Signalling downstream of the photoreceptors under fluctuating shade

### PIFs orchestrate shade avoidance through auxin biosynthesis

Shade avoidance responses involve a group of transcription factors called PHYTOCHROME-INTERACTING FACTORS (PIFs), which activate the transcription of genes needed for the changes in morphology. PIF4, PIF5, and PIF7 make the most important contribution to shade avoidance (Lorrain *et al.*, 2009; Li *et al.*, 2012; Romero-Montepaone *et al.*, 2021). Under sunlight, phyB and cry1 keep PIFs in check. phyB interacts directly with PIFs, recruiting them to nuclear bodies (Pham *et al.*, 2018b; Willige *et al.*, 2021; Chen *et al.*, 2022). Inside the nuclear bodies, phyB facilitates phosphorylation of PIFs, which marks them for ubiquitination and degradation (Lorrain *et al.*, 2008; Leivar *et al.*, 2012; Li *et al.*, 2012; Huang *et al.*, 2018; Pham *et al.*, 2018a; Zhou *et al.*, 2021). Additionally, phyB prevents PIFs from attaching to their target promoters to activate the expression of shade response genes (Qiu *et al.*, 2017; Park *et al.*, 2018; Willige *et al.*, 2021). cry1 also plays a role, interacting with some PIFs and reducing their activity (Ma *et al.*, 2016; Pedmale *et al.*, 2016). When shade falls due to neighbouring plants, the reduced red/far-red ratio and irradiance weaken the inhibition by phyB and cry1. This allows PIFs to escape these controls and activate shade-avoidance genes. Among many other genes, PIFs directly target and enhance the expression of genes involved in auxin synthesis and auxin transport (Hornitschek *et al.*, 2012; Li *et al.*, 2012; Pfeiffer *et al.*, 2014). Neighbour cues elevate the levels of auxin in the growing stem by increasing synthesis in the cotyledons and transport to the growing hypocotyl (Procko *et al.*, 2014). Even the earliest steps of the rapid growth promotion induced by low red/far-red ratios depend on auxin synthesis (Cole *et al.*, 2011).

### Shade-induced feedback loop: COP1 reinforces PIF activity

Shade also initiates a positive feedforward loop. Both phyB and cry1 repress CONSTITUTIVELY PHOTOMORPHOGENIC1 (COP1), which is a component of a CULLIN 4 E3 ligase substrate recognition module (Zhu *et al.*, 2015; Podolec and Ulm, 2018; Ponnu and Hoecker, 2021). In response to shade, COP1 increases its nuclear activity (Pacín *et al.*, 2013), targeting to degradation negative regulators of PIFs such as HYPOCOTYL LONG IN FAR-RED 1 (HFR1) and the DELLA proteins RGA and GAI in hypocotyl cells (Pacín *et al.*, 2016; Blanco-Touriñán *et al.*, 2020). In addition to enhancing auxin synthesis in the cotyledons, PIFs have a local effect in the hypocotyl (Kohnen *et al.*, 2016), and COP1 reinforces this action.

### Persistent shade: refining auxin sensitivity

Under persistent shade, auxin levels return to the values observed before the exposure to neighbour cues, whilst enhanced sensitivity to auxin reinforces the growth response (Hersch *et al.*, 2014; Iglesias *et al.*, 2018; Pucciariello *et al.*, 2018). The stronger sensitivity to auxins is at least partly due to an increase in the abundance of auxin receptor proteins (Pucciariello *et al.*, 2018). Persistent shade enhances the accumulation of PIF4 in vascular tissues, and this pool is involved in the steeper promotion of hypocotyl growth observed under these conditions. *IAA19* and *IAA29* are direct targets of PIF4 that increase their expression in vascular tissues (Pucciariello *et al.*, 2018). These indole acetic acid (IAA) genes promote hypocotyl growth as components of a regulatory loop repressing the expression of *AXR3/IAA17*, which strongly represses hypocotyl growth (Pucciariello *et al.*, 2018). Consistent with this model, the gain-of function *axr2-1/iaa7* mutant, which has enhanced activity of another repressor aux/IAA, lacks petiole growth responses to a persistent low red/far-red ratio (Pierik *et al.*, 2009). The promotion of hypocotyl growth by auxin is mediated by AUXIN RESPONSE FACTOR 6, 7, and 8 transcription factors (Reed *et al.*, 2018), and prolonged shade also increases the abundance of ARF6 in the nucleus of hypocotyl cells (Pucciariello *et al.*, 2018).

### Diurnal gating of shade and auxin responses

As noted in previous paragraphs, daily repeated shade events of 2 h promote hypocotyl growth in Arabidopsis only if they occur late in the photoperiod evening, but are ineffective if they take place consistently in the morning (Sellaro *et al.*, 2012). If instead of shade, the seedlings are exposed daily to the synthetic auxin picloram for 2 h, the dependency of hypocotyl growth on the time of the day is the same. Morning auxin is ineffective, whilst auxin given late in the photoperiod promotes growth, highlighting the correlation of the diurnal sensitivity to shade events and to auxin. Plants require a ‘daytime prep’ (i.e. a prior exposure to light perceived by phyA, cry1, or cry2 photoreceptors) to respond to shade and to auxin (Sellaro *et al.*, 2012). The expression of several PIFs is circadian regulated (Yamashino *et al.*, 2003; Nozue *et al.*, 2007; Niwa *et al.*, 2009), and the circadian clock can influence growth and gene expression responses to neighbour cues (Halliday *et al.*, 2003; Fraser *et al.*, 2021; Martínez-Vasallo *et al.*, 2023, Preprint). Yet, there is no obvious general link between the clock and the pattern of diurnal sensitivity to shade (Sellaro *et al.*, 2012). However, repressing the responsivity to morning shade events requires the morning-expressed transcription factors LATE ELONGATED HYPOCOTYL (LHY) and CIRCADIAN CLOCK ASSOCIATED 1 (CCA1) (Sellaro *et al.*, 2012).

### Mitigating shade responses during lightflecks

The interruption of shade can negatively affect the processes described in the previous paragraphs. Under laboratory conditions, in flickering light (repeated cycles of 1 min of red light and 1 min of far-red light), phyB induces responses to light primarily through PIF sequestration rather than degradation (Park *et al.*, 2018). In seedlings exposed to simulated shade, a single sunfleck of 2 min containing white light and UV-B radiation has no effect on the nuclear abundance of PIF4. However, if these pulses are repeated even with a low frequency (2 min sunfleck, 6 min shade), they provoke a reduction in PIF4 in parallel with the reduction in hypocotyl growth (Belmonte *et al.*, 2024, Preprint). The light-induced reduction in PIF4 nuclear abundance is much faster than its recovery back in shade, helping to amplify the effect of the sunflecks. A significant proportion of the genes with expression repressed by low-frequency sunflecks correspond to PIF4 targets, including genes involved in auxin transport and perception, which are important for the growth response (Belmonte *et al.*, 2024, Preprint).

Shade potentiates key targets of UVR8 activity. As a result of this situation, UV-B perceived by UVR8 is more effective to trigger degradation of PIF4 and PIF5, stabilize HFR1, inhibit the expression of auxin synthesis genes, and inhibit hypocotyl growth in plants grown under low than high red/far-red ratios (Hayes *et al.*, 2014; Tavridou *et al.*, 2020). UVR8 has these effects at least in part by reducing COP1 activity by direct interaction, which outcompetes COP1 signalling targets (Lau *et al.*, 2019; Wang *et al.*, 2024). When shade is interrupted by sunlight, there is an optimum combination of high abundance of UVR8 targets with high UVR8 activation.

The transcription factor ELONGATED HYPOCOTYL 5 (HY5) can compete with PIF4 for its DNA-binding sites (Toledo-Ortiz *et al.*, 2014) and can reduce the expression of *PIF4* (Delker *et al.*, 2014). Low-frequency sunflecks (repeated 2 min sunflecks followed by 6 min shade) are not effective to modify the nuclear levels of HY5 (Belmonte *et al.*, 2024, Preprint). However, sunpatches (daily 2 h interruptions of shade) can increase *HY5* expression and HY5 nuclear abundance, which plays a key role in the repression of auxin signalling and shade avoidance, and the induction of pathways involved in photoprotection under these conditions (Sellaro *et al.*, 2011; Moriconi *et al.*, 2018).

## Concluding remarks

Photoperiod changes with season, but irradiance and spectral composition fluctuate over time scales ranging from months to fractions of a second due to variation in solar elevation, cloudiness, and vegetation interactions, further influenced by wind conditions. Plants face the challenge of extracting meaningful information from this complex light environment. The research community has primarily focused on plant responses under constant light conditions, leaving a significant gap in our understanding of dynamic plant behaviour in their natural fluctuating environment.

Some patterns emerge from recent research about shade-avoidance responses to light fluctuations. First, the response to a light fluctuation of no more than a couple of hours depends on the time within the photoperiod when it occurs. Fluctuations occurring during the final part of the photoperiod tend to be more effective when they are repeated at a similar time on successive days. Second, when the exposure to a fluctuation is repeated, the response can either increase or decrease, as observed under infrequent lightflecks separated by shade, and daily exposures to brief morning shade, respectively. These divergent fates could be linked to the informational value of the fluctuation as only information-carrying variations are useful towards improved fitness. Third, the responses to different cues are not necessarily additive. When *Arabidopsis thaliana* plants are exposed to a sequence of contradictory cues, the impact of light appears stronger than that of shade. For instance, a short exposure to UV in a sunfleck, can block the response to a longer exposure to shadelight. This hierarchy could be inverted in more competitive species. Fourth, whilst plants can respond to even subtle cues from neighbours in the field, they exhibit buffering capacity in their sensory network, filtering out noisy fluctuations. For example, drops in irradiance and red/far-red ratio at photoperiod extremes, caused by atmospheric factors unrelated to competition, have negligible influence on shade avoidance responses.

Our understanding of the underlying molecular machinery that dynamically regulates plant morphology in response to light fluctuations remains in its infancy. While shade avoidance research has yielded valuable insights, focusing solely on new regulatory mechanisms may offer limited progress without considering the precise ecological context in which these mechanisms operate. By incorporating dynamic light fluctuations into future research, both as treatments in controlled experiments and as a cue to be described in natural environments, we will be able to unlock some of the remaining secrets of plant responses in their natural world.

## Supplementary data

The following supplementary data are available at *JXB* online.

Fig. S1. Short wavelength radiation is more diffuse than long wavelength radiation.

Fig. S2. The reduction in irradiance caused by clouds is stronger at longer than at shorter wavelengths.

Fig. S3. Compared with direct radiation, diffuse radiation is proportionally enriched in short wavelengths.

erae334_suppl_Supplementary_Figures_S1-S3
