# Supplementary material for: Making the most of canopy light: shade avoidance under a fluctuating spectrum and irradiance
Source: J Exp Bot. 2024 Aug 5;76(3):712–29. doi: 10.1093/jxb/erae334 (PMC11805590; doi:10.1093/jxb/erae334)
Supplement: erae334_suppl_Supplementary_Figures_S1-S3 [file erae334_suppl_supplementary_figures_s1-s3.pdf]

Supplementary data

Making the most of canopy light: Shade avoidance under a fluctuating spectrum and irradiance.

Romina Sellaro, Maxime Durand, Pedro J. Aphalo and Jorge J. Casal

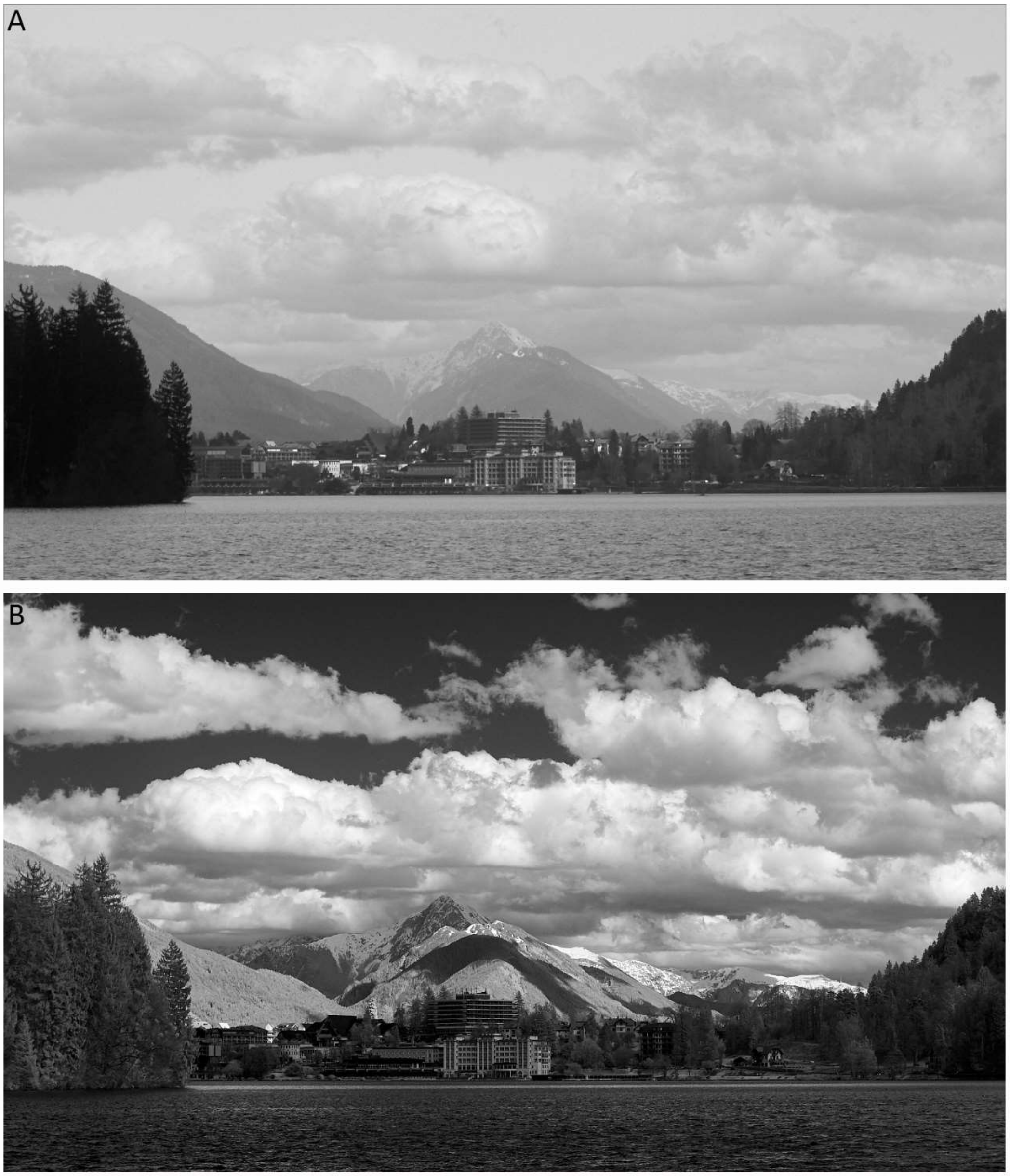

Fig. S1. Short wavelength radiation is more diffuse than long wavelength radiation. Photographs of lake Bled, Slovenia. A. Image taken in ultraviolet-A (UV-A1, 340-400 nm) and (B) in the far red (> 740 nm). The images were acquired within 1 min of each other, using a modified mirrorless camera, with a Bader Venus-U filter and a Heliopan RG-760 filter, respectively.

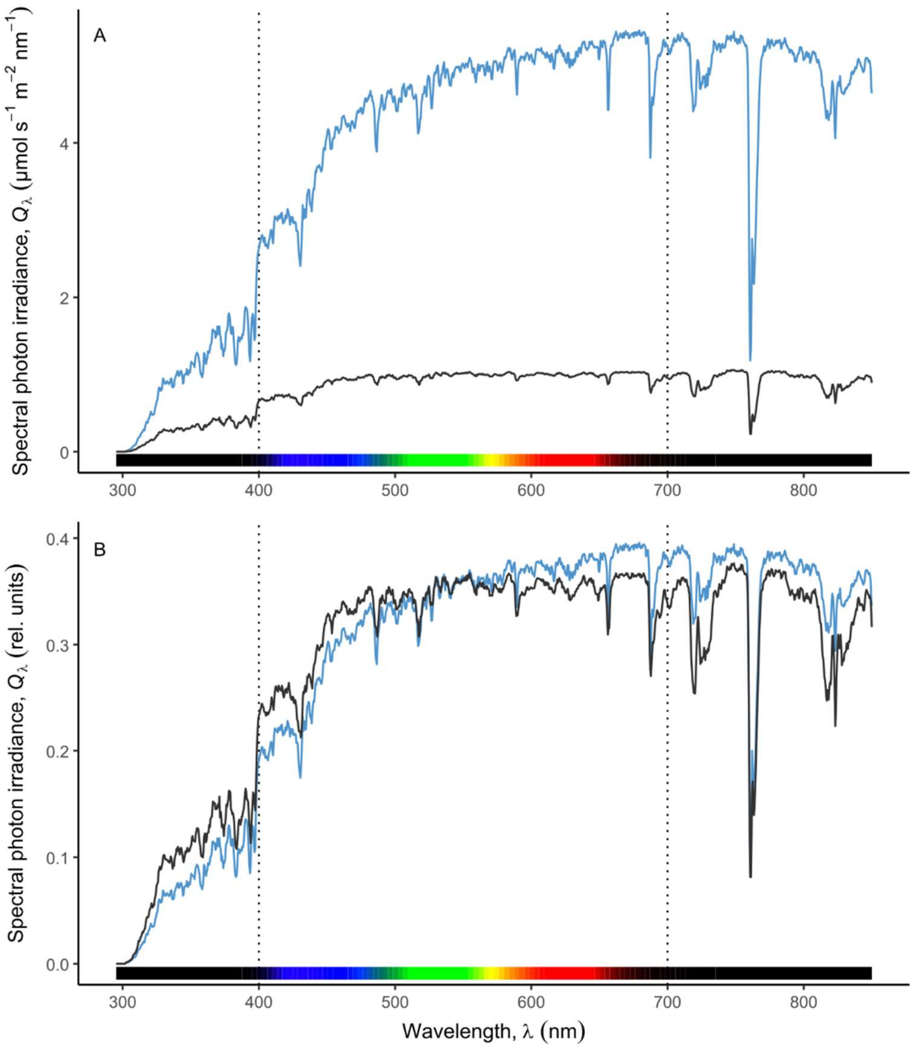

Fig. S2. The reduction in irradiance caused by clouds is stronger at longer than at shorter wavelengths. When the sun is occluded by clouds the relative contribution of skylight to total irradiance increases. Measurements were taken in an open field at noon, on the 28th April (overcast sky, grey line) and the 29th April (clear sky, blue line) 2016, at Lammi Biological Station in southern Finland (61.05 N, 25.04 E). The median curves of 50 spectra are shown as spectral photon irradiance in absolute values (A) or re-scaled to equal PAR of 100 µmol m^{-2} s^{-1} (B). Redrawn from (Durand *et al.*, 2021).

Durand M, Matule B, Burgess AJ, Robson TM. 2021. Sunfleck properties from time series of fluctuating light. Agricultural and Forest Meteorology **308**–**309**, 108554.

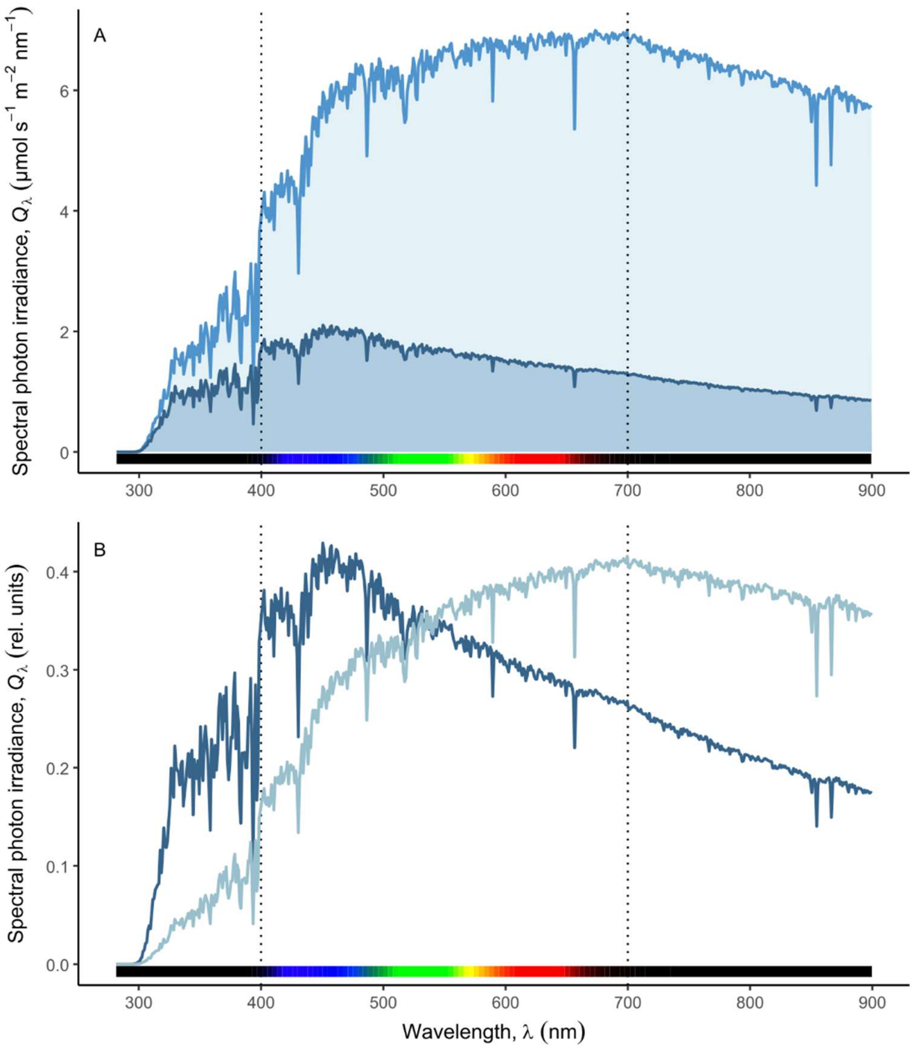

Fig. S3. Compared to direct radiation, diffuse radiation is proportionally enriched in short wavelengths. Daylight spectrum components of diffuse radiation (from sky, dark blue) and direct radiation (from sun, clear blue). Spectral photon irradiance is shown in absolute values (A) or re-scaled to equal PAR of 100 µmol m^{-2} s^{-1} (B). Simulated for clear sky conditions and solar elevation of 60 degrees under typical atmospheric composition for mid latitudes using the TUV radiation transfer model (Bais *et al.*, 2003).

Bais AF, Madronich S, Crawford J, *et al.* 2003. International Photolysis Frequency Measurement and Model Intercomparison (IPMMI): Spectral actinic solar flux measurements and modeling. Journal of Geophysical Research: Atmospheres **108**.
